# Validation and Utility of Drug-Nutrient Interaction and Dietary Supplement Mechanistic Activity in the Natural Medicines Database

**DOI:** 10.1200/OA-24-00062

**Published:** 2024-12-06

**Authors:** Blake O. Langley, Eileen Rillamas-Sun, Yuhan Huang, Amy Indorf, Michael Robles, Rachel Feaster, Lia D'Addario, Isaac J. Ergas, Janise M. Roh, Lawrence H. Kushi, Heather Greenlee

**Affiliations:** ^1^ Fred Hutchinson Cancer Center, Public Health Sciences Division, Seattle, WA; ^2^ University of Washington, School of Public Health, Seattle, WA; ^3^ Fred Hutchinson Cancer Center, Department of Pharmacy, Seattle, WA; ^4^ Kaiser Permanente Northern California, Division of Research, Oakland, CA; ^5^ University of Washington, School of Medicine, Seattle, WA

## Abstract

**PURPOSE:**

The increasing use of dietary supplements by patients with cancer and other chronic diseases requires the systematized review of potential interactions between prescription drugs and nutrients from supplements by health care and clinical research teams. Dietary supplement interaction databases are positioned to fill a gap in quantifying potential risks for patients, although none have been assessed for reliability in data interpretation. The NatMed database, a source for comprehensive reports on mechanistic and safety data for dietary supplement ingredients, was evaluated for use in future investigations.

**METHODS:**

Data from NatMed were retrieved using licensed end points for ingredient monographs with drug-nutrient interactions with doxorubicin across five pharmacokinetic and metabolic pathways, and for ingredient monographs with antioxidant activity. Interactions between dietary supplements and doxorubicin treatment and antioxidant monographs were independently reviewed and characterized by clinical pharmacists. Cohen's K was used to measure interrater reliability and the degree of agreement between pharmacists.

**RESULTS:**

Three hundred fifteen potential interactions with doxorubicin (n = 115 monographs) and 455 other antioxidant ingredients were identified and reviewed by clinical pharmacists. There was substantial to near-perfect agreement for drug-nutrient interactions with doxorubicin (Cohen's K = 0.64-0.85) and for antioxidants (Cohen's K = 0.84). A small proportion of retrieved monographs were not validated by the clinical pharmacists for interactions with doxorubicin (n = 20 occurrences, 6.4%) or for antioxidant activity (n = 28, 6.2%).

**CONCLUSION:**

A high degree of reliability in data on dietary supplement interactions with doxorubicin and mechanisms of action suggests NatMed may be a dependable source of data for future investigators. Additional procedures including independent data validation and use of multiple dietary supplement interaction databases will strengthen the quality of findings in future studies.

## INTRODUCTION

Dietary supplements are increasingly being used by patients both during and after cancer treatment.^1-3^ Despite widespread use, rigorous research on the effects of dietary supplement use during and after active cancer treatment on cancer survival and recurrence is limited and conflicting.^4-6^ Particularly, antioxidants are under close scrutiny for the potential to reduce treatment efficacy by interfering with chemotherapy-induced cellular DNA damage via oxidative stress.^7,8^

**CONTEXT**

**Key Question**
Drug-nutrient interaction databases have not been systematically tested for reliability. Are data on dietary supplement ingredients from a drug-nutrient interaction database consistently interpreted by patients' care teams?
**Knowledge Generated**
Information on drug-nutrient interactions with doxorubicin along five pharmacokinetic and metabolic pathways, and antioxidant activity of dietary supplement ingredients were readily retrieved from the Natural Medicines database. Independent reviews of this information by clinical pharmacists indicated high reliability, and the database was reported to be navigable and user-friendly.
**Relevance *(Y.C. Guerra)***
Evaluations of drug-nutrient interaction database reliability may improve clinician trust and utility in an important resource. Increasing knowledge and confidence of these databases can help enhance communication with patients about the safety and efficacy of dietary supplements during cancer treatment.*
**Plain Language Summary *(M. Lewis)***
The database NatMed, which includes safety information on dietary supplements, can be reliably used to check for interactions with chemotherapy drugs such as doxorubicin and potentially minimize risk to patients.^†^
*Relevance section written by *JCO Oncology Advances* Associate Editor Yanin Chavarri Guerra, MD, MSc, FASCO.
†Plain Language Summary written by *JCO Oncology Advances* Associate Editor Mark Lewis, MD.

Dietary supplement use is also often underreported to health care providers by adults with chronic health conditions, including cancer, and can result in unidentified but clinically significant interactions between drugs, nutrients, and their metabolism.^1,9,10^ The American Medical Association recently released a call for health care personnel to take a more active role in counseling patients on the safety of dietary supplement use.^11^ Yet, limited availability of systematic sources of reliable safety information hinders many clinical teams from answering this call. Therefore, currently available sources of such information need to be evaluated for appropriate use in clinical and research settings and should consider the classes of nutrients found in dietary supplements (eg, antioxidants) in addition to the presence of potential drug-nutrient pharmacokinetic and pharmacodynamic interactions on the basis of patient disease states. Furthermore, researchers must appropriately define concomitant use of dietary supplements to identify plausible drug-nutrient interactions^12,13^ and measure whether the intake of multiple ingredients in a single dietary supplement, or use of multiple dietary supplements simultaneously, commensurately increases the potential for drug-nutrient interactions.^14,15^

**FIG 1.**
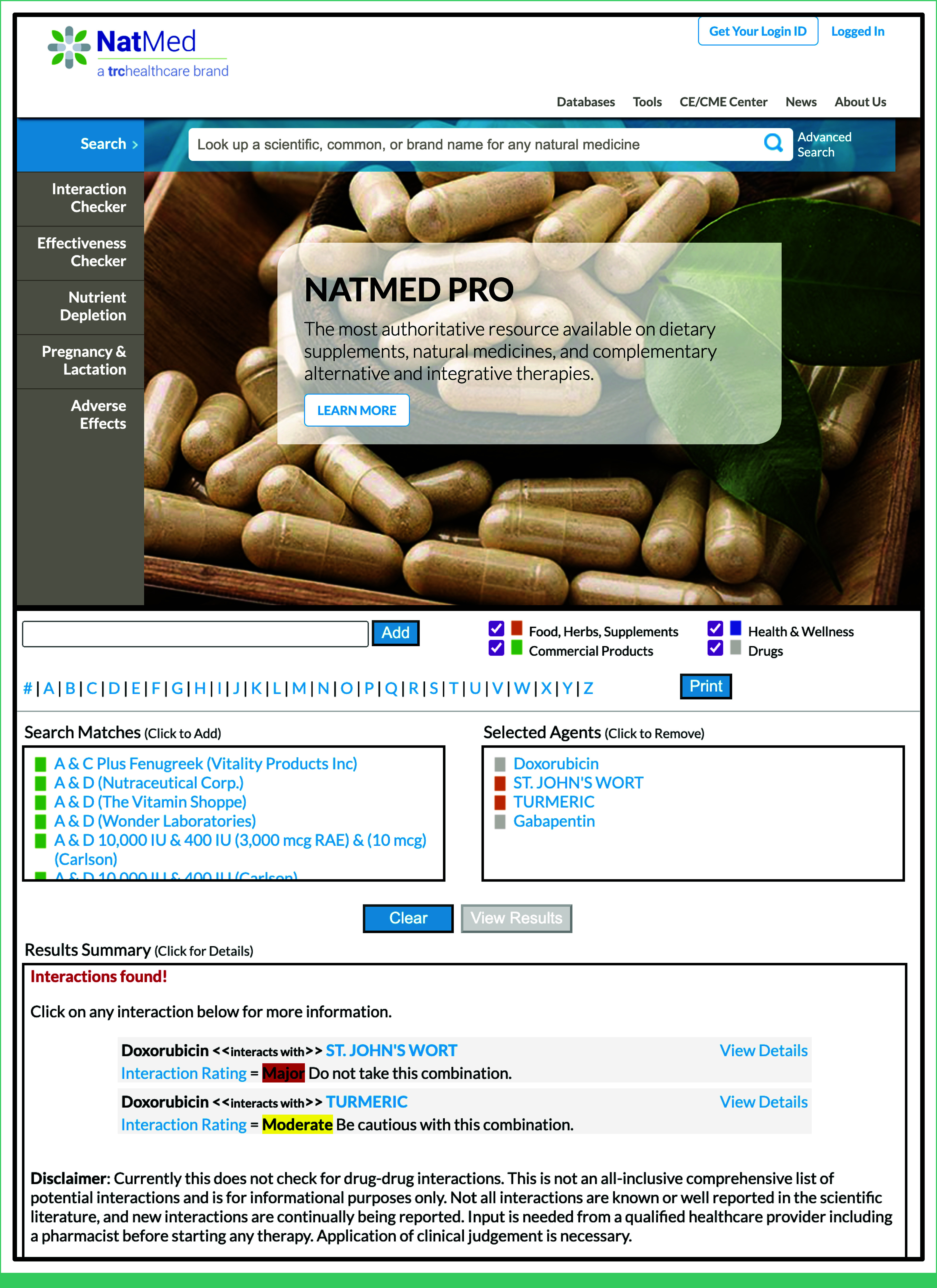
Landing page for NatMed tools (top) with example query of dietary supplement interactions identified with doxorubicin (bottom).^24^ NatMed images printed with permission for the Kaiser Pathways Study by Therapeutic Research Center, LLC, Copyright 2024. All Rights Reserved.

Drug interaction databases, including Drug Interaction Solutions' Drug Interaction Database (DIDB),^16^ Micromedex,^17^ Lexidrug,^18^ and First DataBank's Drug-Drug Interaction Module,^19^ have served as models for dietary supplement interaction databases on drug-nutrient interactions. However, these drug interaction databases have substantial variability^20^ and there remains a paucity of studies validating and confirming drug-nutrient interactions through the use of databases, especially in comparison with the conduct of manual literature searches for clinically relevant research.^21^ NatMed (previously Natural Medicines and Natural Medicines Comprehensive Database)^22,23^ is a comprehensive, peer-reviewed source for both preclinical and clinical data on natural product safety, side effects, efficacy, and drug-nutrient interactions (Fig 1).^25^ On a daily basis, NatMed reviews and evaluates new evidence from multiple bibliographic databases on ingredient mechanism of action and safety for over 1,400 vitamins, minerals, botanicals, nonbotanical supplements, and foods, and relevant updates are incorporated into comprehensive reviews, called *monographs*. NatMed licenses can be secured by individuals or institutions, and is provided as a resource through many academic library systems. For users with a specific type of license agreement, NatMed provides an application programming interface (API) allowing users to retrieve and synthesize available interactions data from individual monographs simultaneously. Thus, NatMed is uniquely positioned to fill the needed gap for researchers investigating outcomes related to dietary supplement use.

This study aimed to evaluate the reliability of NatMed as a source for ingredient antioxidant activity and drug-supplement interactions, focusing on potential drug-supplement interactions with doxorubicin via five prespecified pathways with the potential to affect drug metabolism or efficacy. We hypothesized that a sufficient degree of agreement (no less than moderate agreement) after independent evaluation of output on drug-supplement interactions and antioxidant bioactivity would support the use of NatMed as a reliable central source of data for future research assessing interactions between dietary supplements and cancer treatments. Focusing on the cytotoxic mechanism of doxorubicin, we assessed agreement between clinical pharmacists' evaluation of the accuracy of NatMed using prespecified criteria for all NatMed monographs attributed interactions with doxorubicin and all monographs with antioxidant bioactivity.

## METHODS

### Application Programming Interface

**FIG 2.**
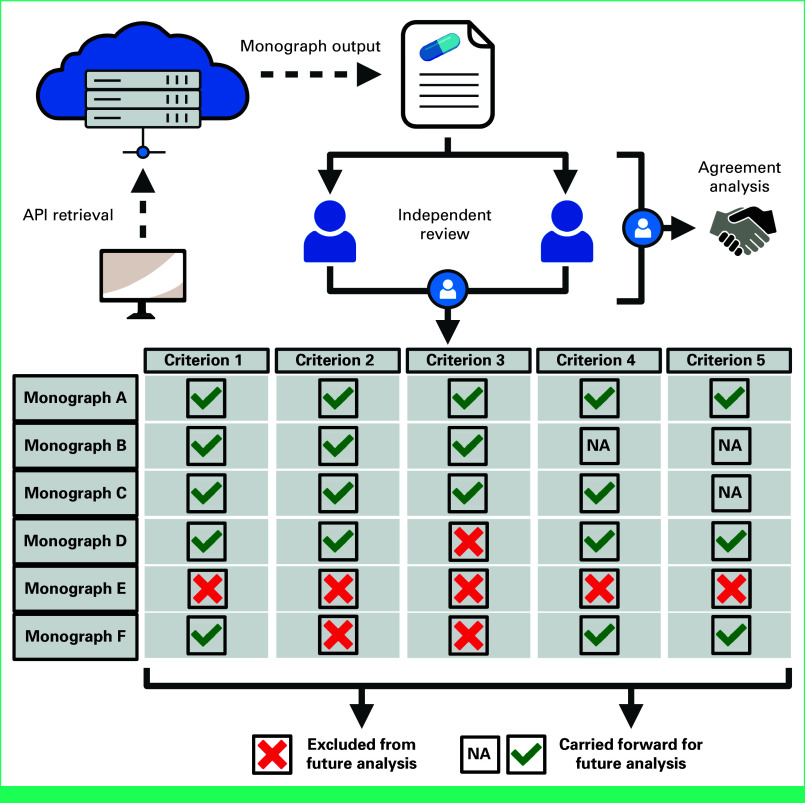
Conceptual model of application programming interface access to NatMed database, independent review and consensus process, and final agreement. API, application programming interface; NA, not applicable. Licensed from Therapeutic Research Center, LLC, Copyright 2024. All Rights Reserved.

Data were retrieved from the NatMed database through a representational state transfer JavaScript Object Notation API (RESTful JSON:API) designed to query specific end points for data retrievals, including an interaction checker end point and monograph end point. The API codes were developed in accordance with guidelines established by Therapeutic Research Center, LLC (TRC),^26^ the parent corporation of NatMed. Retrieval was conducted using *httr* and *rison* packages in R (version 4.0.4). The retrieval, review, and analytical procedures are conceptually depicted in Figure 2.

### Strategy: Identifying Doxorubicin Drug-Supplement Interactions

The NatMed drug interaction checker API end point was used to retrieve all available monographs with doxorubicin interaction attributes on June 21, 2022. Excel files containing the mechanisms of action and drug interaction of the retrieved monographs were downloaded and filtered by searching the following key words in mechanisms of action and drug interaction: antioxidant, antiplatelet, CYP3A4, CYP2D6, and p-glycoprotein. The filtered monographs were subsequently grouped to five, nonmutually exclusive metabolic and pharmacokinetic pathways with known potential to interact with doxorubicin: antioxidant mechanisms,^27^ antiplatelet mechanisms,^28^ cytochrome P450 3A4 (CYP3A4) interactions,^29^ cytochrome P450 2D6 (CYP2D6) interactions,^29^ and p-glycoprotein interactions.^30^

Two clinical pharmacists were provided standardized Microsoft Excel spreadsheets to independently evaluate data from identified monographs for (1) activity within monograph, (2) validity of source evidence from human or preclinical data, (3) validation of data in NatMed monograph report by expert opinion after evaluating available data, (4) drug-drug interaction (DDI) classification (none, minor, moderate, or major), and (5) directionality of interaction (inhibitor, inducer, or mixed/undetermined). For all pathways except antioxidant mechanisms, the pharmacists provided information about clinical relevance of interaction. For the CYP3A4, CYP2D6, and p-glycoprotein pathways, the pharmacist provided information about directionality (ie, whether the ingredient behaves as an inducer or inhibitor). Choice categories available to reviewers for each retrieval can be found in Table 1.

**TABLE 1.** Criteria and Options for Clinical Pharmacist Reviewers to Characterize Dietary Supplements on the Basis of Mechanisms of Action: Antioxidant, Antiplatelet, CYP3A4, CYP2D6, and P-Glycoprotein

Criteria	Option	Antioxidant	Antiplatelet	CYP3A4	CYP2D6	P-Glycoprotein
Presence of mechanism in monograph	1. Yes 2. No	X	X	X	X	X
Human *v* in vitro data available in monograph	1. Yes (human) 2. No (preclinical/in vitro)	X	X	X	X	X
Agreement with NatMed and API results on the basis of available evidence	1. Yes 2. No	X	X	X	X	X
Clinical relevance of interaction	1. None 2. Mild 3. Moderate 4. Severe	NA	X	X	X	X
Directionality of interaction	1. Inhibitor 2. Inducer 3. Multidirectional or undetermined	NA	NA	X	X	X

Abbreviations: API, application programming interface; CYP2D6, cytochrome P450 2D6; CYP3A4, cytochrome P450 3A4; NA, not applicable.

### Strategy: Identifying Antioxidant Mechanisms of Action

Anticipating our future investigation will include dietary supplements with antioxidant activity not related to potential interactions with doxorubicin, the NatMed monograph API end point was used to retrieve all available monographs and respective attributed mechanisms of action (eg, antioxidant, anti-inflammatory, or immunomodulatory) on October 24, 2023. All retrieved monographs were first sorted by mechanism of action categories (eg, analgesic, antimicrobial, and immunomodulatory) and then filtered for antioxidant activity. Three clinical pharmacists were provided standardized Microsoft Excel spreadsheets to independently evaluate data from identified dietary supplement monographs for (1) inclusion of antioxidant activity within monograph, (2) availability of human data as source evidence within the monograph, and (3) validation of data in NatMed monograph report by expert opinion after appraising available data. As monographs with antioxidant activity and interactions with doxorubicin were already reviewed, these monographs were deduplicated and removed before this review.

### Statistical Approach

A third reviewer compared responses from the clinical pharmacists' reviews, flagged discordant responses, and returned the data to the clinical pharmacists for discussion and final agreement. If final agreement was not achieved in the second review, adjudication was facilitated by the independent reviewer. Unweighted Cohen's Kappa (Cohen's K) was used to assess interrater reliability for the decisions in each retrieval using *irr* package in R (version 4.3.2). In retrieval 1 (doxorubicin drug-supplement interactions), interrater reliability was assessed across all five pathways and for each metabolic and pharmacokinetic pathway with respect to interactions with doxorubicin. In retrieval 2 (antioxidant mechanisms of action), interrater reliability was assessed for all monographs with antioxidant activity, including monographs with interactions to doxorubicin. K-values for level of agreement and reliability were set according to established thresholds.^31^

Standardized criteria were established to validate monograph interaction properties and antioxidant activity (Fig 2). Monographs were validated when pharmacists reached consensus for both, (1) applicable activity reported within the monograph (ie, antioxidant or antiplatelet) and (2) pharmacists' validation of data in the monograph on the basis of expert opinion after evaluating available data. Additional consensus-based characteristics were applied to monographs after this validation to source data type, DDI classification, and directionality of interaction. When reviewers were unable to reach consensus on specific monographs, the monograph was deemed unvalidated with the plan to be excluded from any subsequent analyses.

### Pharmacists' Feedback on Platform Use

Pharmacists were prompted to provide feedback in a semistructured manner on the usability and reliability of the NatMed platform when reviewing monographs, and for any suggested changes to the interface that would support clinical decision making. Specific prompts were provided for pharmacists to consider the effectiveness of NatMed reporting layouts, and their ability to critically evaluate the applicability and validity of the sources of evidence. Feedback was collected via written qualitative text. Qualitative data were assessed for emergent themes present across the participating pharmacists to provide context to end-consumer challenges and opportunities for improvement for future researchers using the platform.

### Ethics

This investigation did not involve human participants and therefore did not require review by an institutional review board. All data used were obtained from NatMed and TRC.

## RESULTS

### Doxorubicin Drug-Supplement Interactions

One hundred fifteen (n = 115) unique NatMed monograph records were retrieved with potential interactions between dietary supplements and doxorubicin. Most monographs had more than one mechanism of action attributed to potential interactions with doxorubicin (88.7%, n = 102 monographs). Monographs with CYP3A4 activity tended to have additional mechanisms found in other pathways (Appendix Table A1). A small number of monographs were attributed with activity across all five mechanistic pathways (5.2%, n = 6). The distribution of monographs within each mechanism of action pathway can be found in Table 2.

**TABLE 2.** Distribution of Monographs With Interaction Between Doxorubicin and One or More Dietary Supplement Ingredients for 115 Monographs in the NatMed Database

Domains	Frequency, No. (%)
1 domain only	18 (15.7)
Antioxidant	4 (3.5)
Antiplatelet	1 (0.9)
CYP3A4	13 (11.3)
CYP3D6	0 (0.0)
P-glycoprotein	0 (0.0)
2+ domains	102 (88.7)
Antioxidant + antiplatelet	44 (38.3)
Antioxidant + CYP3A4	70 (60.9)
Antioxidant + CYP2D6	23 (20.0)
Antioxidant + p-glycoprotein	26 (22.6)
Antiplatelet + CYP3A4	47 (40.9)
Antiplatelet + CYP2D6	16 (13.8)
Antiplatelet + p-glycoprotein	18 (15.7)
CYP3A4 + CYP2D6	33 (28.7)
CYP3A4 + p-glycoprotein	28 (24.3)
CYP2D6 + p-glycoprotein	12 (10.4)
3+ domains	65 (56.5)
Antioxidant + antiplatelet + CYP3A4	38 (33.0)
Antioxidant + antiplatelet + CYP2D6	13 (11.3)
Antioxidant + antiplatelet + p-glycoprotein	15 (13.0)
Antioxidant + CYP3A4 + CYP2D6	23 (20.0)
Antioxidant + CYP3A4 + p-glycoprotein	22 (19.1)
Antioxidant + CYP2D6+ p-glycoprotein	9 (7.8)
Antiplatelet + CYP3A4 + CYP2D6	16 (13.9)
Antiplatelet + CYP3A4 + p-glycoprotein	15 (13.0)
Antiplatelet + CYP2D6+ p-glycoprotein	7 (6.1)
CYP3A4 + CYP2D6 + p-glycoprotein	12 (10.4)
4+ domains	26 (22.6)
Antioxidant + antiplatelet + CYP3A4 + CYP2D6	13 (11.3)
Antioxidant + antiplatelet + CYP3A4 + p-glycoprotein	13 (11.3)
Antioxidant + antiplatelet + CYP2D6 + p-glycoprotein	6 (5.2)
Antioxidant + CYP3A4 + CYP2D6 + p-glycoprotein	9 (7.8)
Antiplatelet + CYP3A4 + CYP2D6 + p-glycoprotein	7 (6.1)
All domains	6 (5.2)

NOTE. Proportions reported as a percentage of all monographs reviewed (n = 115).

Abbreviations: CYP2D6, cytochrome P450 2D6; CYP3A4, cytochrome P450 3A4.

Across all pathways, initial review resulted in substantial to near-perfect agreement between pharmacists (K = 0.81, n = 1,356 observations; Table 3). The highest level of agreement was for monographs with CYP2D6 activity (K = 0.85, n = 220 observations), while the lowest level of agreement was for monographs in the antioxidant mechanism of action pathway (K = 0.64, n = 246 observations). On the basis of final assessment of monographs between pharmacists, retrieval over-reported potential interactions with doxorubicin 6.4% of following pharmacist review (n = 20 of 315 observations; Appendix Table A1). No remaining disagreements remained after one round of consensus review.

**TABLE 3.** Agreement on Dietary Supplement Interaction Properties With Doxorubicin by Independent Review

Domain of Assessment	Antioxidant	Antiplatelet	CYP3A4	CYP2D6	P-Glycoprotein
Total monographs, No.	82	55	101	44	33
PharmDs agree NatMed states interaction, No. (%)	69 (84.1)	52 (94.5)	100 (99.0)	32 (72.7)	33 (100)
PharmDs agree with NatMed interaction assessment, No. (%)	75 (91.5)	50 (90.9)	90 (88.6)	39 (88.6)	29 (87.9)
Human data available in NatMed summary, No. (%)	67 (81.7)	51 (92.7)	87 (86.1)	35 (79.5)	30 (90.9)
Degree of interaction (DDI classification), No. (%)	—	53 (96.4)	100 (99.0)	44 (100)	30 (90.9)
Agreement on interaction type (inhibitor/inducer), No. (%)	—	—	95 (94.1)	44 (100)	29 (87.9)
Cohen's K for interrater reliability	0.64	0.82	0.85	0.85	0.82
Accuracy of API retrieval for identifying interaction, %	92.7	92.7	99.0	86.4	90.9

Abbreviations: API, application programming interface; CYP2D6, cytochrome P450 2D6; CYP3A4 cytochrome P450 3A4; DDI, drug-drug interaction; PharmD, licensed clinical pharmacist.

### Antioxidant Mechanism of Action

Five hundred thirty-seven (n = 537) monographs were retrieved with antioxidant activity, of which 455 unique NatMed monograph records were reviewed, the 82 monographs with potential interactions with doxorubicin notwithstanding. Initial review resulted in strong agreement between the clinical pharmacists (K = 0.84, n = 1,365 observations; Table 4). No remaining disagreements remained after one round of consensus review. A small number of monographs from retrieval 92 were not validated for antioxidant activity by the clinical pharmacists (n = 28, 6.2%), or were monographs for therapeutic diets or interventions (n = 9, 2.0%). Strong agreement was achieved when the reviewed records from both retrievals were merged (92.4% overall agreement, K = 0.84, n = 1,581 observations).

**TABLE 4.** Agreement Between Pharmacists on Dietary Supplement Antioxidant Properties

Domain of Assessment	Antioxidant
Total No. of monographs	455
PharmD agrees NatMed states interaction, No. (%)	434 (95)
PharmD agrees with NatMed interaction, No. (%)	413 (89)
Human data available in NatMed summary, No. (%)	413 (91)
Cohen's K for interrater reliability	0.84

Abbreviation: PharmD, licensed clinical pharmacist.

### Pharmacist Feedback

From feedback provided by two pharmacists, the NatMed platform was a simple, easy-to-use, and navigable platform to review drug-nutrient interactions and monograph mechanisms of action. NatMed was also comparable in structure and function to other drug interaction databases, providing a familiar end-user experience. Each pharmacist also reported the regular need to perform their own detailed analysis of available data to ascertain the exact clinical relevance of source evidence; however, the amount of evidence available was generally sufficient to appropriately guide clinical decision making. This additional scrutiny was considered time-consuming, and data were predominantly on the basis of preclinical, pharmacokinetic studies with limited reports on pharmacodynamic or drug-disease interactions. Furthermore, limited data on the dose-dependence of interactions relative to the concentrations that may be reported by patients restricted the translation of findings to clinical applications.

## DISCUSSION

This investigation evaluated the reliability of data retrieved from NatMed on drug-nutrient interactions and dietary supplement mechanisms of action via an independent review by clinical pharmacists. The two retrievals using API compiled data on dietary supplement ingredient monographs with attributed interactions with doxorubicin across five metabolic and pharmacokinetic pathways, and with attributed antioxidant activity. Independent review of monographs by clinical pharmacists achieved substantial to near-perfect agreement for both retrievals, suggesting data retrieved from NatMed is reliably interpreted between users when reviewed in a structured manner. Our review validated the NatMed API end points were dependable tools to identify ingredient mechanistic pathways of interaction systematically and accurately with 93.7% overall accuracy. Qualitative feedback suggests the end-user interface is intuitive, but a relative scarcity of data on pharmacodynamic and drug-disease interactions are impediments to research applied to larger, clinical data sets.

Antioxidant supplements are especially concerning for potential interactions with, and reduced efficacy of, oxidative cancer treatments.^7,8^ A review of over 30 randomized controlled trials examining antioxidant supplement use during chemotherapy reported over half of the patients taking antioxidant supplements experienced a decrease in chemotherapy-related toxicity.^32^ A separate review of 19 randomized controlled studies of over 1,500 patients demonstrated no reduction in chemotherapy efficacy (ie, overall survival and complete/partial response or remission) associated with the use of antioxidant dietary supplements.^33^ By contrast, an ancillary study to a phase III trial in 1,134 women with early-stage breast cancer found antioxidant supplement use during chemotherapy treatment of cyclophosphamide, doxorubicin, and paclitaxel suggested a possible increased risk of breast cancer recurrence (hazard ratio [HR], 1.41 [95% CI, 0.98 to 2.04]) and all-cause mortality (HR, 1.83 [95% CI, 1.15 to 2.92]) relative to nonuse.^3^ This study also reported greater risks of poorer clinical outcomes with nonantioxidant supplements, specifically increased risk of vitamin B12 supplementation with disease-free survival (HR, 1.83 [95% CI, 1.15 to 2.92]) and overall survival (HR, 2.04 [95% CI, 1.22 to 3.40]), and with iron supplementation with breast cancer recurrence (HR, 1.37 [95% CI, 1.20 to 2.67]). Thus, while dietary supplements are generally considered safe in the general population, conservative guidance on their use during and after chemotherapy in oncology patients has been recommended, necessitating further investigation of specific types of dietary supplements (ie, antioxidants) and their possible health effects among cancer survivors.

To our knowledge, ours is the first study focused on determining the appropriateness of a dietary supplement interaction database for future researchers to use when assessing drug-nutrient interactions. The standardized management and review of data provided a rigorous and thorough understanding of the validity of database-driven approaches to exploring potential drug-nutrient interactions and dietary supplement ingredient activity. End points for APIs to query data within NatMed provided a rapid and efficient approach to access and appraise specific topics related to our research questions. Our process further solicited end-user feedback to ascertain operational and data drawbacks for future research teams to consider when developing similar procedures.

Our study also had some limitations. Not unique to NatMed, there are risks that data within drug interaction databases may be incomplete,^20^ inaccurately structured in relation to the data query,^34^ or inconsistently interpreted by teams focusing in other areas related to drug-supplement interactions.^35^ Much of the data reported in the reviewed monographs were from preclinical studies, highlighting the need for confirmation of findings in human studies.^21^ However, the high interrater reliability suggests NatMed is a dependable platform for interaction analyses related to specific pharmaceuticals. With these considerations, future investigators of drug-nutrient interactions on the basis of dietary supplement data should use more than one database when exploring drug or dietary supplement interactions, ensure independent review and data validation is conducted before formal analysis, and should consider reporting if drug-supplement interactions were based solely on preclinical data. Finally, formal comparison of dietary supplement interaction databases is needed to identify differences in reported evidence.^20^

In an American Medical Association article proposing reforms to current dietary supplement regulations, Richardson et al^11^ acknowledged the vital role of physicians and pharmacists in counseling patients about dietary supplement use. Yet barriers, such as clinicians' inadequate knowledge about dietary supplements, limited time with patients at appointments, and lack of scientific evidence about the effects of dietary supplement use on cancer treatment, hamper the ability of clinicians to have effective conversations with their patients.^36^ The findings of our analysis offer support and confidence to clinicians that the NatMed database is a sound source of information on the reliability and accuracy of antioxidant activity and doxorubicin interactions in dietary supplements. Developing and providing up-to-date trainings for clinicians on how to effectively navigate the NatMed database could help improve knowledge and efficiency in finding safety and side-effect information. If feasible, clinical oncology pharmacists trained in integrative medicine can be leveraged to support physicians in checking safety, appropriateness, and interactions with cancer treatments and can aid in patient counseling. Indeed, a study at Memorial Sloan Kettering Cancer Center reported that use of an integrative medicine pharmacist to support patient-provider communication on the use of Chinese herbs during cancer treatment was feasible, safe, and had high patient satisfaction.^37^ Additionally, such a pharmacist has been supporting the integrative medicine clinics at the Fred Hutchinson Cancer Center by checking safety and drug-drug and drug-disease interactions with supplements, creating and implementing guidelines around supplement use, and educating clinicians.^38^

A logical next step for researchers is to explore associations between dietary supplements and long-term clinical outcomes, leveraging patient-reported dietary supplement data from longitudinal cohorts on the basis of data from interaction databases. Our team agrees with the call to action by Wolf et al^39^ to develop frameworks by which investigators may assess data from large cohorts on the exact risks posed by potential dietary supplement interactions. NatMed uses a standardized process for mapping patient-reported dietary supplements to individual ingredients, including a large array of commercial products with respective ingredients. Our study supports NatMed as a dependable source of data on dietary supplement mechanisms of action and drug-supplement interactions, with high reliability and consistency for interpreting data on mechanisms of action. Therefore, we anticipate NatMed will be a suitable standard for future investigations into ingredient safety and interactions using clinical trial, cohort, and registry data, which have monitored dietary supplement use.

In conclusion, dietary supplements are a potential source for interactions with both pharmaceuticals and other dietary supplements. NatMed, a comprehensive dietary supplement interaction database, was found to have a high degree of reliability in data retrieved on dietary supplements on the basis of interactions with doxorubicin and mechanisms of action after independent review by clinical pharmacists. Intuitive end-user interface and bulk data retrieval through established API end points position NatMed as a dependable source of data for research studies on the associations between the use dietary supplement and clinical outcomes from observational or other clinical trials. Future studies using such databases should employ additional procedures to confirm data and extracted variables specific to the research question, which may not be retrievable in a systematic manner.

## APPENDIX

**TABLE A1.** Overall Accuracy of Application Programming Interface Retrieval for Characterization of Interaction With Doxorubicin

Mechanism of Action	Monographs, No.	No Interaction Found, No.	Accuracy, %
Antioxidant	82	6	92.7
Antiplatelet	55	4	92.7
CYP3A4	101	1	99.0
CYP2D6	44	6	86.4
P-glycoprotein	33	3	90.9

Abbreviations: CYP2D6, cytochrome P450 2D6; CYP3A4, cytochrome P450 3A4.
